# Outpatient Waiting Time at Vietnam Health Facilities: Policy Implications for Medical Examination Procedure

**DOI:** 10.3390/healthcare8010063

**Published:** 2020-03-20

**Authors:** Dinh-Hoa Nguyen, Dinh-Van Tran, Hoang-Long Vo, Hao Nguyen Si Anh, Thi-Ngoc-Ha Doan, Thi-Huyen-Trang Nguyen

**Affiliations:** 1 Institute of Orthopedic Trauma, Viet Duc University Hospital, Hanoi 100000, Vietnam; ndhoavietducspine@gmail.com; 2 Social Affair Department, Viet Duc University Hospital, Hanoi 100000, Vietnam; 3 Department of Surgery, Hai Duong Medical Technical University, Hai Duong 170000, Vietnam; 4 Department of Neurosurgery I, Viet Duc University Hospital, Hanoi 100000, Vietnam; tranvanpttk@gmail.com; 5 Institute for Preventive Medicine and Public Health, Hanoi Medical University, Hanoi 100000, Vietnam; doanthingocha.hmu@gmail.com; 6 Department of Public Health, Thang Long University, Hanoi 100000, Vietnam; trangnth@thanglong.edu.vn

**Keywords:** outpatient waiting time, health facility, Vietnam, examination procedure

## Abstract

Our study aims to measure outpatient waiting times at Vietnam health facilities according to the socioeconomic characteristics. We employed the 2015 Vietnam District and Commune Health Facility Survey which was a cross-sectional study designed by the World Bank in collaboration with the Vietnam Health Strategy and Policy Institute. This survey was designed to be representative of six provinces (Dien Bien, Hanoi, Binh Dinh, Dak Lak, Dong Nai, and Dong Thap) drawn from six distinct geographical regions of Vietnam. Data from 4949 outpatients at district hospitals (DHs) and 1724 outpatients at commune health centers (CHCs) were extracted for final analysis. We recorded average outpatient waiting times of 32.58 min at DHs and of 11.58 min at CHCs. Four hundred and forty-five outpatients at DHs (9.0%) and 720 those at CHCs (42.8%) were examined immediately (waiting time = 0 min). Outpatient waiting times were various in six distinct geographical regions. With an investigation according to several socioeconomic characteristics, significant differences in outpatient waiting times were observed at both two levels of health facilities as measured by province, age, self-reported health status, patient’s wealth, ethnicity, and health insurance. *Conclusions.* Outpatient waiting times from arrival at health facility until receiving care were significantly distinct amongst two health facility levels, revealing longer at DHs compared to at CHCs. There was significantly higher proportion of outpatients examined immediately at CHCs compared to at DHs. Our study suggests that, vulnerable populations, with longer outpatient waiting time, should be dealt with in appropriate models towards each medical facility according to key socioeconomic factors to contribute to simplify the process of medical examination and treatment for outpatients.

## 1. Introduction

Patient satisfaction has been measured as a crucial parameter in the assessment of health care quality. In which, the waiting time is a primary index to be included in the evaluation of patient satisfaction from a psychological perspective, particularly, previous studies reported that, the longer the patient waited for the examination, the more their dissatisfaction was [1,2,3]. Hence, the waiting time is also known as one of important contributors to access the quality of patient services [4]. The time interval the patient needs to wait before being examined by a health worker or experiencing health service was considered as the patient waiting time [3,5,6]. However, for the outpatient waiting time, there are two core aspects of the waiting time of an outpatient who receives medical treatment without being admitted to a hospital. It firstly is that the waiting time of patient was mentioned by the physicians from entering the clinic until the doctor to the end of the examination with the conclusion for the patient’s health condition. The other aspect was known as the waiting time from their arrival at health facility until being taken care of or entering the examination room. Notably, the second aspect is more concerned because of its relevance to the hospital’s subsequent medical activities [7].

Outpatient waiting time varies between countries worldwide. A study using a universal sampling method at a primary care clinic located in a large district of Gombak (located in the state of Selangor, Malaysia) reported that the average patient waiting time from registration to be examined by a doctor was 41 min [8]. In Atlanta (the US), the average waiting time of patient was found to be approximately 60 min, while the average figure for a study in Michigan was 188 min [9]. In a descriptive cross-sectional study conducted in the general outpatient department of a tertiary health institution in northern Nigeria, more than 60% of patients experienced the waiting time of 90–180 min before being seen by a clinical doctor [10]. Nevertheless, the determinants that influenced the waiting times have not been disaggregated according to the characteristics of the patient, and these studies were not representative of a large sample of the population, as well as are focusing on only patients in a treatment health facility/clinic. 

The long waiting time of patient might lead to public health-related troubles, including impaired access to care, interruption of hospital work patterns, and patient dissatisfaction. Especially in the socioeconomic context of a developing country such as Vietnam, the overload of patients in hospitals is prevalent, which gives rise to extended waiting times [11,12]. This country has, currently, been facing great challenges to find out comprehensive solutions towards the patient waiting time at health facilities [11,12]. Previous studies of the waiting time have only stopped at individual hospitals, and have not been included to socioeconomic factors in the correlation with patient waiting time. Importantly, seeking evidence-based solutions to enhance the quality of healthcare services is always put in the context of the interactions of socioeconomic characteristics. Thus, our paper along with the desire is contributing to fill the broad literature using outpatient waiting time as a measure of access to medical care at Vietnam’s health facilities. The aim of this study was to measure the outpatient waiting time at Vietnam health facilities based on socioeconomic characteristics, thereby, formulating a strategy to reduce waiting time based on focusing on priority key socioeconomic factors.

## 2. Methods 

### 2.1. Data Source

**Figure 1:**
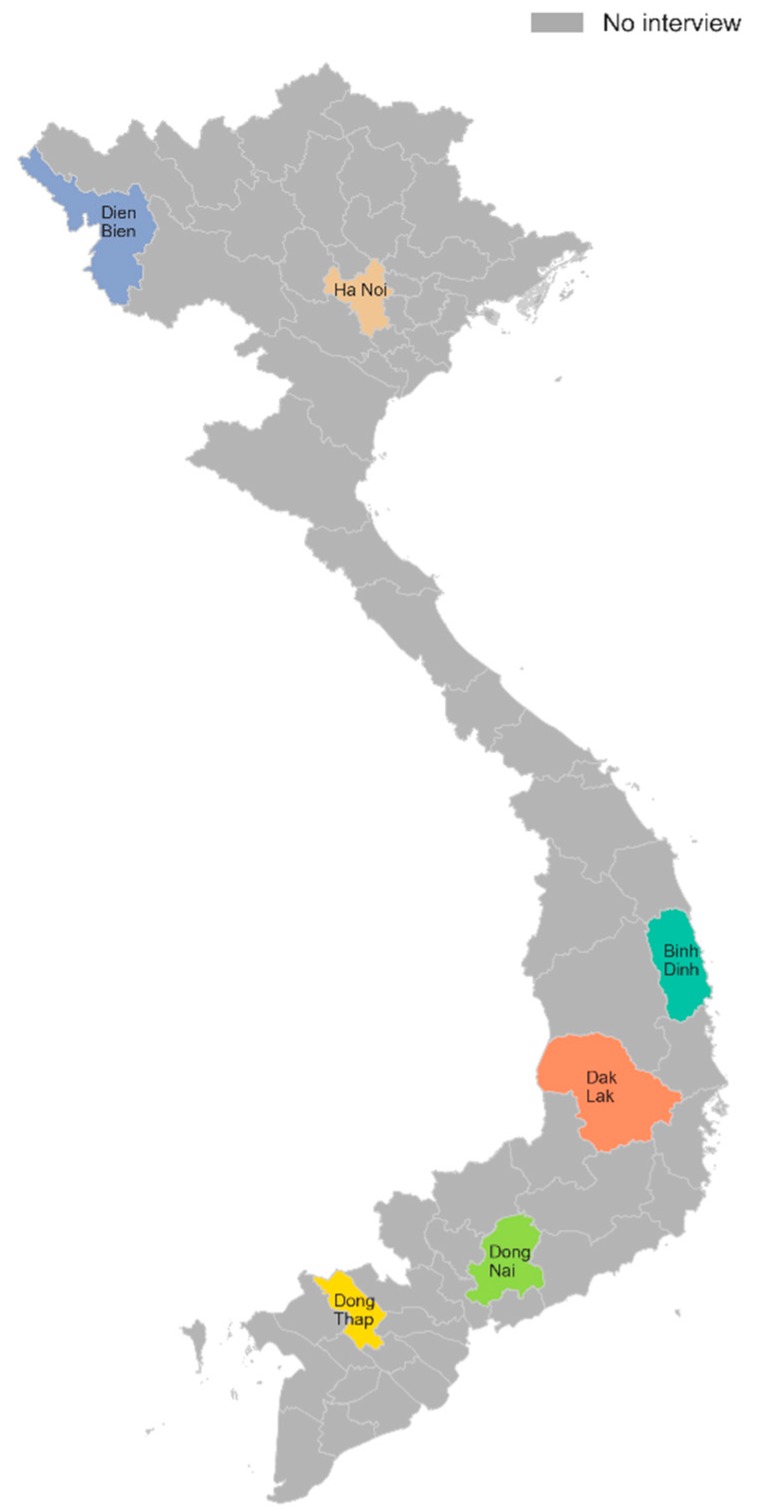
The provinces selected in the 2015 Vietnam District and Commune Health Facility Survey.

This study used secondary data from the World Bank survey “2015 Vietnam District and Commune Health Facility Survey” which was conducted with a quantitative cross-sectional survey between 17 May 2015 and 1 July 2015. The Health Strategy and Policy Institute (HSPI) of the Vietnamese Ministry of Health in collaboration with the World Bank carried out this study. This survey’s objectives were to provide the information about health facility preparedness, doctor characteristics, and patient experiences. The survey was designed to be representative of six provinces (Dien Bien, Hanoi, Binh Dinh, Dak Lak, Dong Nai, and Dong Thap) drawn from six distinct geographical regions of Vietnam (Figure 1). The 2015 Vietnam District and Commune Health Facility Survey was carried with the same locations as the 2015 household survey to ensure the linkage in analyzing the associations amongst the health seeking behavior and the quality of local providers. The sample of the health facility survey were commune health centers (CHCs) and district hospitals (DHs) locating in the communes and districts, respectively that were corresponding with the selected enumeration areas (clusters) in the household survey. This is a nationally representative survey covering a broad range of the information of CHCs and DHs in all communes and districts, as well as the information of patients participating in these facilities (Vietnam health care system is organized across four administrative levels of health establishments: Central level, provincial level, district level, and commune level. In which, Vietnam’s grassroots health care system includes commune health stations belonging to commune level and district hospitals belonging to district level. National and central hospitals are located in the country’s major urban centers and provide highly-specialized tertiary care for referrals from provincial hospitals. Provincial and district hospitals offer secondary care, accepting referrals for more specialized treatment from the lower levels. Commune health centers deliver basic primary care and prevention services on an outpatient basis. Detailed descriptions of the 2015 Vietnam District and Commune Health Facility Survey can be found in the survey report [13]. 

### 2.2. Study Subjects

We extracted data for outpatients at DHs and CHCs. A total of 78 DHs and 246 CHCs in selected provinces were included in this study. At each DH, all outpatients were examined by two selected doctors. At each CHC, all outpatients were examined by doctors/assistant doctors (an assistant doctor was known as a physician assistant). All interviews with patients at both DHs and CHCs were conducted face-to-face by the field researchers who were carefully trained to become more familiar with the questionnaire contents, sampling, and approaches of data collection. A total of 4949 patients at DHs and 1724 patients at CHCs were extracted from the 2015 Vietnam District and Commune Health Facility Survey for final analysis [13].

### 2.3. Sampling Design

This survey used the multi-stage sampling method with three stages: 

Stage 1: Six provinces (Dien Bien, Hanoi, Binh Dinh, Dak Lak, Dong Nai and Dong Thap) were selected as a “typical” of six distinct geographical regions of Vietnam based on criteria of provincial average income per capita and provincial poverty rates.

Stage 2: Cluster selection was applied with the Probability Proportional to Size (PPS) method used. The sample clusters were selected based on the sampling frame of six provinces used in the 2014 Intercensal survey (stratified by rural/urban for each province). The household lists and maps were checked, reviewed, and updated to use in this survey. 

Stage 3: Household was selected with using the Systematic Randomly Selection (SRS) method. In each cluster, 25 households were systematic randomly selected from the list of households.

### 2.4. Variables

#### 2.4.1. Outcome Variable

In this study, the outcome variable was outpatient waiting time. Outpatient waiting time referred to the total number of minutes that each patient spent waiting from their arrival at health facility (DH and CHC) until being taken care of [13]. 

#### 2.4.2. Socioeconomic Variables

The socioeconomic variables we selected in this study were age group (<15/15–60/≥60), gender (male/female), ethnicity (Kinh/nonKinh), self-reported health status (bad or very bad/normal/good or very good), the first visit to DH/CHC referred to the first time the patients were seeking medical care at DH/CHC (yes/no), health insurance (insured/uninsured), provinces (Dien Bien, Hanoi, Binh Dinh, Dak Lak, Dong Nai, and Dong Thap), and socioeconomic status. The socioeconomic variables selected in this paper were because, in their medical records in Vietnam’s health facilities, these are considered as the main information originally declared by the patient or their family at the time of coming to the hospitals/health centers for the examination. In addition, these are also given as initial variables that patients need to answer in the general information section of the questionnaire in the “2015 Vietnam District and Commune Health Facility Survey”.

### 2.5. Measurement of Socioeconomic Status

Socioeconomic status comprised five groups from poorest to richest. The “poorest” and “richest” were defined as quintile 1 and quintile 5 determined by the wealth index of the patients. The principal component analysis method was applied to compute the wealth index based on the patients’ household assets selected from the durable list of Vietnam Living Household Standard Survey in 2014. The assets were listed as washing machine, (bath) water heater, computer, refrigerator, gas/magnetic cooker, cell phone, electric (rice/pressure) cooker, desk/chair/long bench/dressing table, motorbike, and color TV [13].

### 2.6. Data Analysis

Both descriptive and analytical statistics were used to address the main aim of the study. Mean, median, and interquartile range (IQR) were used for overall outpatient waiting times at DHs and CHCs. We calculated outpatient waiting times with mean, standard deviations, frequencies, and percentages according to each selected socioeconomic characteristics (age group, gender, ethnicity, self-reported health status, the first visit to DH/CHC referred to the first time the patients were seeking medical care at DH/CHC, health insurance, provinces, and socioeconomic status). Measurement of outpatient waiting times by selected characteristics is expressed by different testing in mean values between groups and association evaluation. First, the Kruskal–Wallis test (for comparison of three groups or over) and Mann–Whitney test (for comparison of two groups) were applied to compare the difference of the average outpatient waiting times amongst characteristics’ subgroups. Then, the linear relationships between outpatient waiting times and selected characteristics were estimated with multivariate linear regression. Further, to better illustrate the overall picture of outpatient wait times nationwide, a dot distribution map was employed to show the average outpatient waiting times at DHs and CHCs in six separate provinces. All statistical analysis was performed with Python 3.6—programming language (Python Software Foundation) (https://www.python.org/about/). The level of statistical significance was considered at *p* < 0.05.

### 2.7. Research Ethics

This paper was based on data extracted from the 2015 Vietnam District and Commune Health Facility Survey. After having stated the use purposes, all users were allowed to access the dataset and the report with all identifying information removed [13]. The survey obtained informed consent from patients at DHs and CHCs before administering a face-to-face interview with standard questionnaire. All users of the data are encouraged to share the research findings.

## 3. Results

**Figure 2:**
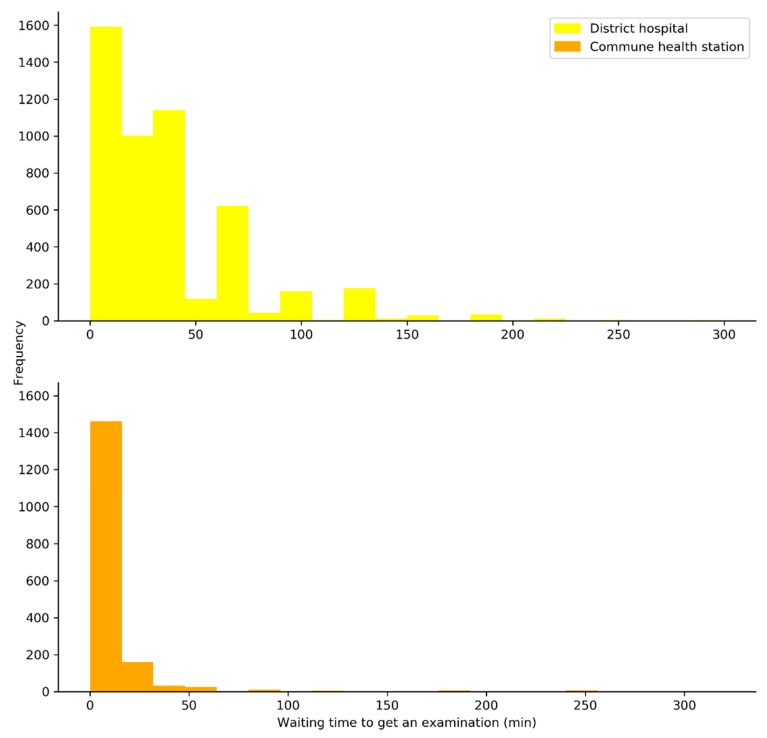
Distribution of the outpatient waiting times in district hospitals (DHs) and commune health centers (CHCs).

Figure 2 illustrates the distribution of outpatient waiting times in DHs and DHCs. Average outpatient waiting times from arrival at health facility until receiving care were 32.58 min at DHs and 11.58 min at CHCs. The median figures for outpatient waiting times were 20 min (IQR: 10) at DHS and 5 min (IQR: 30) at CHCs. Four hundred and forty-five outpatients at DHs and 720 those at CHCs were examined immediately (waiting time = 0 mins), with the proportion accounted for 9% and 42.8%, respectively.

**Figure 3:**
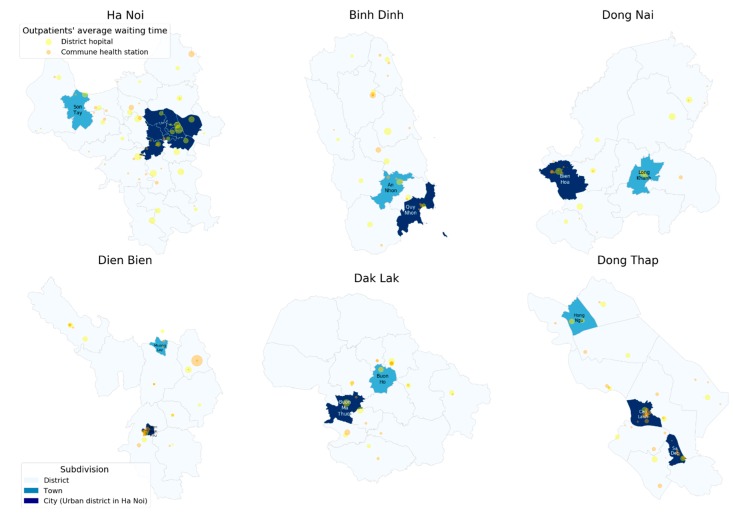
A dot distribution map of average outpatient waiting times at commune health stations and district hospitals in each province according to commune health stations and district hospitals.

Average outpatient waiting times at DHs and CHCs in each category by selected socioeconomic characteristics are presented in Table 1. At DHs, the older the age group was, the longer the outpatient waiting time was. Outpatient waiting time at DHs decreased with better self-reported health status and higher socioeconomic group. Kinh ethnicity (33.0 ± 35.6 min) experienced longer outpatient waiting time at DHs than nonKinh people (26.7 ± 30.1 min), vice versa amongst ethnic populations at CHCs (8.3 ± 14.4 and 28.8 ± 64.2 min). Outpatient waiting time at CHCs was much higher among patients having health insurance (12.3 ± 30.8 min) compared to their counterparts (2.8 ± 5.0 min). The longest outpatient waiting times at DHs among patients living in Hanoi and Dong Nai were 36.0 ± 43.5 and 37.8 ± 37.1 min, respectively, while, at CHCs, the longest outpatient waiting time among those residing in Dien Bien was 35.5 ± 70.9 min. There were such significant differences in average outpatient waiting times amongst subgroups of above groups. Figure 3 provides an overview on the difference amongst six separate provinces, which were clearly observed with the dot distribution of outpatient waiting times corresponding to the distribution of the health facilities. 

Table 2 shows the result of multivariate linear regression of associations between outpatient waiting times and socioeconomic characteristics. At DHs, the incidence rate ratio of outpatient waiting time among the Kinh patients was 1.19 (95% CI 1.07–1.33) higher than the incidence rate ratio for nonKinh, while Kinh patients at CHSs had 0.28 (95% CI 0.22–0.37) lower incidence rate ratio of outpatient waiting time than nonKinh. The patients who the first time were seeking medical care at DHs had 1.21 (95% CI 1.09–1.34) higher incidence rate ratio of outpatient waiting time compared to their counterparts. 

## 4. Discussion

In the present study, each province selected in the 2015 Vietnam District and Commune Health Facility Survey represents a specific geographical area in Vietnam. The local or regional impacts of culture and economy on the access to health care services might lead to various waiting times of patients before having a doctor’s meeting in provinces that belong to Vietnam’s six regions. Importantly, the healthcare system at health facilities has been overloaded for a long time, resulting in deterioration not only in the patient’s satisfaction towards healthcare services but also in stretching out the state of undergoing pain, distress, or hardship [14,15]. All if this could be linked with extended waiting time, one of the initial aspects of access to health care services. With regards to the outpatient waiting time considered from a patient’s arrival to meeting clinical staffs, the study found that the average outpatient waiting times were 32.58 min at DHs and 11.58 min at CHCs. The average waiting times at both DHs and CHCs were significantly lower in this study than previous studies in Vietnam, 50.41–42.05 min at Viet Duc Hospital [16], 198 min at Trung Vuong emergency hospital [17], 4–6 h at Bach Mai hospital. It could be explained that, while we reported the outpatient waiting times at health facilities (DHs and CHCs) directly under districts and communes, most previous studies focused only on provincial/central hospitals that were considered the highest health facility level in Vietnam. The patient overload was prevalent in most hospitals at all levels, especially at the central-and provincial-level hospitals, which leaded to lengthening the waiting times of patients. A further reason was that, the number of patients coming to health facilities at lower-level hospitals (district/commune hospitals) was much less than that of the central-and provincial-level hospitals. Therefore, the waiting time of the patient to see a doctor could be shortened once the number was less patients.

We believe that the age group research in this paper is of interest as Vietnam has now entered the period known as the “Golden Population Structure” which means that for every two or more people working, there is only one dependent person [18]. We considered the elderly as people who are at least 60 years old. By looking at the outpatient waiting times by different age groups, we found that one of the striking results was that, at the hospitals located at districts, the outpatient waiting time of the elderly was actually longer than the nonelderly. Our finding is in line with a previous Vietnam study at a highest-level hospital [19]. In fact, the Vietnamese elderly seeking medical care rely on their children or relatives for help to get the easier approach. Therefore, the process of medical examination and treatment at district hospitals not yet focusing effectively on vulnerable groups such the elderly may be an appropriate explanation for our finding. Importantly, self-reported health status was reported to also be associated with age, with older people more likely to report poor health than younger people [20,21]. We found that the outpatient waiting time at DHs were shortened with better self-reported health status. The fact that, in the context that the proportion of Vietnamese elderly people living alone reported to be increasing over time [18,22], which requires more specific social support services at health facilities towards the elderly to shorten waiting times in the medical examination process. Policymakers should consider planning in the distribution of waiting time according to appropriate age groups based on this finding, thereby, prioritizing the Vietnamese elderly population in waiting toward medical examination and treatment processes. 

Socioeconomic status based on the patients’ household assets are less studied in association with waiting time. We considered socioeconomic status as a key characteristic in this study. In both DHs and CHCs, we found that the difference in socioeconomic status for the waiting times of outpatients for an examination with the doctor. Especially, at grassroots medical health facilities known as CHCs, the patients with higher socioeconomic status had a decreased average waiting time. In general, in Vietnam, an extensive network of CHCs comprised of administrative health units organized much simpler compared to higher-level health facilities. Therefore, richer patients attending the lowest health service network known as CHCs might be more likely to pay money to increase their chances of getting quicker access to medical care, while this issue is more tightened in higher-level health facilities. This insight probably implied an existing problem at the grassroots level in Vietnam, which was an inequity in waiting for a medical examination between rich and poor.

Ethnicity emerged as a prominent factor in this study, revealing the difference in the outpatient waiting time between two health facility levels. At the hospitals of district level, Kinh ethnicity experienced longer waiting time at DHs than nonKinh people. The reason might be that, in several selected districts, the local governments have created favorable conditions for ethnic minority people (nonKinh) to receive medical examination and treatment at all district hospitals without having to carry out the transfer procedures. This result might be an initial success in health care policy to be implemented in the direction of prioritizing to solve health problems for ethnic minority populations [23,24]. At the lowest level of Vietnam health facility (CHCs), we revealed that the outpatient waiting time was much longer for the nonKinh than for the Kinh. In fact, most minority groups attending at CHCs were those the first to visit for a doctor’s meeting, so they might be not familiar with the health examination procedure at health facilities. Another explanation was that, several ethnic minority populations could not speak Vietnamese, hence, facing difficulties in communicating with commune health staffs. 

Ensuring rationality and equality between patients with health insurance and those not having health insurance is one of the general principles that set out in Decision No. 1313/QD-BYT of Vietnam Ministry of Health [25]. As Vietnamese law has no restrictions on health insurance together with the fact that better techniques and famous doctors are at high-level medical facilities, many patients sought high-level medical facilities for examination and treatment rather than low-level ones, which was given because of better techniques and famous doctors at high-level medical facilities [26]. We found that, at CHCs, those having health insurance (12.3 ± 30.8 min) had to wait for their turn much longer compared to those not having (2.8 ± 5.0 min), conversely, for a previous study at a central surgical hospital (Viet Duc Hospital) [16]. The explanation may be the difference in the level of the health facilities. Previously, the authors reported that the waiting times of patients were at a central hospital—the highest administrative level of health establishment in Vietnam, in which extended waiting times related to complex administrative procedures [16], while the difference among those with and without health insurance in our study was observed at CHSs known as the grassroots health facility. In fact, CHCs do not have complex administrative procedures for the patients, as well as do not face patient overload because the locals in general tend to skip their local health facilities to seek treatment at central hospitals located in the urban centres [27]. Although there existed the difference in the outpatient waiting time between the insured group and the uninsured group, patients at CHCs generally did not take much time to wait for the examination. Further studies are needed to explore factors associated with the patient waiting times in both insured and uninsured patients, such as administrative procedures, people’s beliefs and attitudes towards the use of health insurance, and ways to receive patients from commune health workers.

While the major strength of this study was the use of the nationwide representative survey data, several important limitations needed considering once interpreting the results. First, the 2015 Vietnam District and Commune Health Facility Survey, the data source for this paper, was based on the self-reported information of patients and no validation of the provided information was done from other objective sources. Secondly, this study existed the limitations of a cross-sectional design, and, in particular, the information of the criteria adopted for the multi-stage sampling of the sampling design was not available in the official report “2015 Vietnam District and Commune Health Facility Survey”. Third, we could not access the local’s beliefs and attitudes towards the access to the medical services at different-level health facilities, which might serve as confounding factors to outpatient waiting time. Finally, Vietnam has been experiencing changes and major challenges in the health sector for many years, importantly contributing to waiting times of patient related to the medical examination procedure. However, the available report along with dataset of the Vietnam District and Commune Health Facility Survey has not been updated until the present time. 

## 5. Conclusions

Findings from this study have showed distinct outpatient waiting times (the total number of min that each patient spent waiting from their arrival at health facility until being taken care of) between DHs and CHCs. Notably, there existed a significant difference in the proportion of outpatients who were examined immediately amongst two health facilities, estimated at 9% and 42.8%, respectively. Investigation of outpatient waiting times in the Vietnamese population based on socioeconomic characteristics, the paper found that significant differences in outpatient waiting times remained existent in district hospitals and commune health centers as measured by province, age, self-reported health status, patient’s wealth, ethnicity, and health insurance. In particular, at grassroots medical health facilities known as CHCs, patients who had health insurance, and with lower socioeconomic status had to wait for their turn significantly longer compared to their counterparts. At district hospitals, extended outpatient waiting times were found among patients that belonged to an elder age group and with worse self-reported health status. Current findings are relevant for the providers as the responsibility is on them to ensure patient satisfaction. They should initiate a workable waiting time assessment model in outpatients at the operational level. The practical implication we want to suggest is that the initial report on the initial socioeconomic characteristics of the patient at the visit should be integrated into the process of allocating the patient flow to the clinic in the most optimal way. 

## Figures and Tables

**Table 1:** Average outpatient waiting time according to selected socioeconomic characteristics grouped by health facility.

Characteristics	District Hospital (*n* = 4949)			Commune Health Center (*n* = 1724)		
	*n*	Outpatient’s Average Waiting Time (min) (Mean ± SD)	*p*-Value	*n*	Outpatient’s Average Waiting Time (min) (Mean ± SD)	*p*-Value
Age group			<0.001 ***			0.41
<15	1625	25.7 ± 28.5		438	10.3 ± 27.0	
15–60	2081	34.1 ± 35.7		765	12.1 ± 34.1	
≥60	1243	39.0 ± 40.5		521	11.8 ± 24.7	
Gender			0.16			0.14
Male	2217	31.3 ± 33.3		693	9.2 ± 21.7	
Female	2732	31.6 ± 36.7		1031	13.2 ± 34.1	
Ethnicity			<0.001 ***			<0.001 ***
NonKinh	408	26.7 ± 30.1		276	28.8 ± 64.2	
Kinh	4541	33.0 ± 35.6		1448	8.3 ± 14.4	
Self-reported health status			<0.001 ***			0.20
Bad or very bad	2106	34.6 ± 38.2		715	11.4 ± 28.9	
Normal	2709	31.4 ± 32.8		967	11.8 ± 30.9	
Good or very good	134	24.9 ± 31.3		42	9.7 ± 16.1	
First visit			0.38			0.02 *
No	4454	32.2 ± 34.8		1607	11.9 ± 30.5	
Yes	495	35.7 ± 38.7		117	7.4 ± 16.2	
Health insurance			0.43			<0.001 ***
Insured	4728	32.4 ± 35.1		1593	12.3 ± 30.8	
Uninsured	221	36.2 ± 38.4		131	2.8 ± 5.0	
Socioeconomic status			<0.001 ***			<0.001 ***
1st quintile (poorest)	1017	34.3 ± 33.1		334	21.3 ± 50.9	
2nd quintile	1054	30.3 ± 31.2		388	13.6 ± 33.0	
3th quintile	928	33.9 ± 37.2		468	9.0 ± 15.6	
4th quintile	960	32.7 ± 35.5		197	6.5 ± 12.2	
5th quintile (richest)	990	31.8 ± 38.9		337	6.1 ± 11.9	
Provinces			<0.001 ***			<0.001 ***
Ha Noi	1110	36.0 ± 43.5		280	9.1 ± 19.8	
Dien Bien	315	21.3 ± 25.9		217	35.5 ± 70.9	
Binh Dinh	575	31.8 ± 33.9		139	13.3 ± 18.6	
Dak Lak	769	31.0 ± 34.9		240	8.0 ± 11.5	
Dong Nai	870	37.8 ± 37.1		317	2.6 ± 6.2	
Dong Thap	1310	30.1 ± 26.9		531	9.6 ± 12.7	

*, ***: Significant at 0.05 and 0.001, respectively.

**Table 2:** Multivariate linear regression of the outpatient waiting time according to selected socioeconomic characteristics.

Characteristics	Comparison	District Hospital		Commune Health Station	
		IRR	95% CI	IRR	95% CI
Age		1.01	1.00–1.01	1.01	1.00–1.01
Gender	Female vs. Male	1.05	0.99–1.12	1.17	0.96–1.40
Ethnicity	Kinh vs. NonKinh	1.19	1.07–1.33	0.28	0.22–0.37
First visit	Yes vs. No	1.21	1.09–1.34	0.85	0.59–1.25
Self-reported health status	Bad or very bad vs. Normal	1.05	0.99–1.12	1.16	0.971.40
	Good or very good vs. Normal	0.83	0.67–1.00	1.17	0.76–1.98

IRR: Incidence rate ratio; CI: Confidence interval.
